# Supplementary material for: The Complete Mitochondrial Genome of Pilumnopeus Makianus (Brachyura: Pilumnidae), Novel Gene Rearrangements, and Phylogenetic Relationships of Brachyura
Source: Genes (Basel). 2022 Oct 25;13(11):1943. doi: 10.3390/genes13111943 (PMC9690104; doi:10.3390/genes13111943)
Supplement: Supplementary file 1 [file genes-13-01943-s001.zip › genes-1937301-supplementary.pdf]

Table S1 List of Brachyuran species with their GenBank accession numbers.

superfamily	family	species	Size	Accession.no
Pilumnoidea	Pilumnidae	*Pilumnus vespertilio*	16222	MF457402
		Echinoecus nipponicus	16173	NC_039618
		Pilumnopeus makianus	15863	OM461360
Leucosioidea	Leucosiidae	*Myra affinis*	15349	MW192449
		Pyrhila pisum	15516	NC_030047
Portunoidea	Portunidae	*Charybdis hellerii*	15913	MW228889
		Thalamita spinicarpa	15783	MW309529
		Thalamita sima	15625	MW309528
		Charybdis japonica	15748	MW446892
		Charybdis annulata	15747	MF198249
		Charybdis granulata	15774	MW446891
Gecarcinucoidea	Gecarcinucidae	*Somanniathelphusa yangshanensis*	17206	OL693861
		Somanniathelphusa hainanensis	17212	OL693860
		Somanniathelphusa bawangensis	17208	MN737135
		Somanniathelphusa boyangensis	17032	NC_032044
		Esanthelphusa dugasti	19437	OL693858
		Esanthelphusa keyini	17172	OL693859
Potamoidea	Potamidae	*Longpotamon depressum*	16537	NC_057478
		Nanhaipotamon hongkongense	15318	NC_057474
		Tenuilapotamon latilum	19294	MW788029
		Terrapotamon thungwa	16156	MW697087
		Aparapotamon similium	19236	NC_053821
		Neilupotamon xinganense	16965	MN117718
Xanthoidea	Panopeidae	*Rhithropanopeus harrisii*	15902	MW446897
	Xanthidae	*Macromedaeus distinguendus*	15710	NC_057473
		Etisus dentatus	15884	NC_054248

		Etisus anaglyptus	16435	NC_042208
		Atergatis floridus	16180	NC_037201
		Atergatis integerrimus	15924	MG786939
		Leptodius exaratus	15716	MF198250
		Matuta victor	15782	NC_053638
Calappoidea	Matutidae	*Matuta planipes*	15751	MK281334
		Ashtoret lunaris	15807	NC_024435
	Epialtidae	*Scyra compressipes*	16415	MW451225
	Oregoniidae	*Oregonia gracilis*	15737	NC_057204
Majoidea		*Chionoecetes opilio*	16067	MT335860
		Maja squinado	16598	NC_035425
	Majidae	*Maja crispata*	16592	NC_035424
		Leptomithrax sp.	16608	MG571272
Cancroidea	Cancridae	*Cancer pagurus*	42736	NC_051962
		Metacarcinus magister	39658	NC_050675
		Grapsus albolineatus	15578	NC_057301
		Metopograpsus quadridentatus	15517	NC_038178
	Grapsidae	*Metopograpsus frontalis*	15587	NC_042152
		Pachygrapsus marmoratus	15406	NC_039109
		Grapsus tenuicrustatus	15858	NC_029724
		Pachygrapsus crassipes	15652	NC_021754
		Cardisoma armatum	15586	NC_057477
	Gecarcinidae	*Gecarcoidea lalandii*	15575	NC_057475
		Cardisoma carnifex	15597	NC_039105
		Chasmagnathus convexus	15107	NC_052834
		Varuna litterata	16247	NC_056882
Grapsoidea		*Cyclograpsus intermedius*	16184	NC_050045
		Pseudohelice subquadrata	16898	NC_042685
	Varunidae	*Neoeriocheir leptognathus*	16143	NC_041211
		Metaplax longipes	16305	NC_040976
		Gaetice depressus	16288	NC_038179
		Varuna yui	15915	NC_037155
		Helicana wuana	16359	NC_034995
		Perisesarma bidens	15641	NC_051868
		Chiromantes eulimene	15894	NC_047209
	Sesarmidae	*Chiromantes haematocheir*	15899	NC_042142
		Chiromantes dehaani	15917	NC_041212
		Parasesarma affine	15638	NC_039990
		Tubuca arcuata	15727	NC_058243
Ocypodoidea	Ocypodidae	*Ocypode stimpsoni*	15557	NC_046797
		Austruca lactea	15659	NC_042401

		Cranuca inversa	15677	NC_039111
		Scopimera intermedia	16252	NC_057476
	Dotillidae	*Dotilla wichmanni*	15600	NC_038180
		Ilyoplax deschampsi	15460	NC_020040
		Macrophthalmus abbreviatus	16322	NC_057472
	Macrophthalmidae	*Macrophthalmus pacificus*	17226	NC_046039
		Macrophthalmus japonicus	16170	NC_030048
	Mictyridae	*Mictyris longicarpus*	15548	NC_025325
Outgroup		*Pagurus nigrofascia*	15423	NC_042412
		Pagurus gracilipes	16051	LC222534
